# One-Step Crank-Nicolson Direct-Splitting Algorithm with Enhanced Absorption to Evaluate Low-Pressure Discharge for Satellite Sensors in Outer Space

**DOI:** 10.3390/s23031085

**Published:** 2023-01-17

**Authors:** Yangjing Wang, Yongjun Xie, Pu Su, Haolin Jiang, Peiyu Wu

**Affiliations:** 1 School of Electronic and Information Engineering, Beihang University, Beijing 100191, China; 2 Beijing Key Laboratory of Microwave Sensing and Security Applications, Beihang University, Beijing 100191, China; 3 Xi’an Institute of Space Radio Technology, Xi’an 710100, China; 4 Shenzhen Institute of Beihang University, Shenzhen 518000, China; 5 School of Electronic & Information Engineering, Nanjing University of Information Science and Technology, Nanjing 210096, China

**Keywords:** Crank-Nicolson Direct-Splitting (CNDS), Finite-Difference Time-Domain (FDTD), Low-Pressure Discharge, Perfectly Matched Layer (PML), Satellite Sensors

## Abstract

Low-pressure discharge causes air ionization resulting in performance degeneration or failure for the satellite sensors in outer space. Here, a one-step Crank-Nicolson Direct-Splitting (CNDS) algorithm is proposed to evaluate the electrical behavior of satellite sensors under the low-pressure discharge circumstance. To be more specific, the CNDS algorithm is proposed in the Lorentz medium, which can accurately analyze the ionized air and generated plasma. Higher order perfectly matched layer (PML) is modified in the Lorentz medium to efficiently terminate the unbounded lattice. It can be concluded that the proposed algorithm shows entire considerable performance in the low-pressure discharge evaluation. The proposed PML formulation behaviors enhanced absorbing performance compared with the existing algorithm. Through the experiments, it can be observed that the low-pressure discharge phenomenon causes performance variation, which shows a significant influence on the satellite sensors. Meanwhile, results show considerable agreement between the simulation and experiment results which indicates the effectiveness of the algorithm.

## 1. Introduction

For the satellite sensors in outer space, special space characteristics, which include passive inter-modulation, multipactor, and so on, significantly affect the entire performance [1,2,3]. Among them, low-pressure discharge can be regarded as one of the most urgent and valuable challenges in the development of satellite sensors [4]. Although the satellite sensors are designed to work in a vacuum, the gas which is occurred by glue for connection causes the generation of a low-pressure environment [5]. In such circumstances, high power, which is employed for excitation, ionizes the gas resulting in the occurrence of discharge [6]. Most importantly, the low-pressure discharge phenomenon leads to the occurrence of multipactor phenomenon in most circumstances which leads to performance degeneration, component failure or even satellite scrapping [7]. Although the multipactor phenomenon has raised concern, the low-pressure discharge phenomenon still needs to be further investigated and studied.

When a high power source excites the components, the appearance of the low-pressure discharge phenomenon results in gas ionization. Such a condition leads to the forming of plasma which shows a significant influence on electrical behavior. In order to efficiently analyze the such condition, plasma simulation is regarded as one of the most important elements. According to the physics of plasma, it can be expressed by the Lorentz medium in the finite-difference time-domain (FDTD) algorithm [8]. The Lorentz medium can be solved by the piecewise linear recursive convolution scheme (PLRC), trapezoidal recursive convolution scheme (TRC), JE convolution (JEC) scheme and so on [9,10,11,12]. Among them, it has been testified that the PLRC method shows the most considerable accuracy [13].

The FDTD algorithm, proposed by Yee, shows potential in broadband problems with complex mediums [14]. By applying the FDTD algorithm to simulate sensors with a large number of fine details, extremely long simulation duration is occurred due to the limitation of stability conditions. The conventional FDTD algorithm is a time-explicit algorithm whose stability is limited by the Courant--Friedrichs--Levy (CFL) condition [15]. This means that a constant relationship is established between the time step and mesh size. If the CFL stability condition is broken, the conventional explicit algorithm is no longer stable. In order to alleviate the such condition, unconditionally stable algorithms are proposed to overcome the stable condition [16,17,18]. Most unconditionally stable algorithms are based on the sub-step procedure, which means splitting the entire equation into several steps to obtain the final results. Such condition limits the entire performance, including the efficiency and accuracy of the unconditionally stable algorithms. The Crank--Nicolson (CN) scheme solves Maxwell’s equations through the one-step procedure. The original CN algorithm can merely solve problems in one dimension [19]. By applying it to multi-dimensions, large sparse matrices should be calculated, resulting in an expensive calculation. In order to alleviate such conditions, approximate CN schemes are introduced to accurately solve Maxwell’s equations [20,21]. It should be noted that two-dimensional approximate CN algorithms cannot be directly expanded to three dimensions [22]. Thus, three-dimensional approximate CN algorithms are carried out, including the approximate-factorization-splitting (AFS) and direct-splitting (DS) schemes [23,24]. However, the CNAFS algorithm must solve nine matrices in a single update cycle which results in a significant increment in simulation duration and calculation resources [25]. The CNDS algorithm solves six matrices in a full update cycle resulting in improvement in terms of effectiveness compared with the CNAFS algorithm [26].

In the full-wave simulation method, an adequate boundary condition must be employed to terminate the unbounded lattice. The perfectly matched layer (PML) is regarded as the most powerful absorbing boundary condition [27]. The original PML formulation is a split-field scheme that results in the degeneration of efficiency and absorption [28]. In order to alleviate such a condition, the unsplit-field formulation is introduced into the PML formulation, including the stretched coordinate (SC) and complex-frequency-shifted (CFS) schemes [29,30]. It shows advantages in absorbing the late-time reflections and reducing the low-frequency evanescent waves. However, wave reflections at the low-frequency still show unacceptable levels in some circumstances. The reason is that the low-frequency propagation waves cannot be efficiently absorbed [31]. In order to alleviate such drawbacks, a higher-order formulation is employed, which can be implemented by multiplying the stretched coordinate terms together into a single term. The original higher-order formulation holds six auxiliary variables which affect the efficiency and absorption [32]. Alternative higher-order formulation with four auxiliary variables is introduced to improve the entire performance, which has been extensively employed in massive problems [33,34,35,36].

Although several schemes are introduced based on the CNDS and CNAFS schemes to solve open regions problems, medium dependent characteristic of the PML formulation results in non-general formulation inside different materials [37,38,39,40]. Thus, the existing formulation cannot be applied to the solving of the low-pressure discharge for satellite sensors with complex frequency-dispersion Lorentz medium. By applying these schemes directly to such calculation, the unmatched impedance between the domain and boundaries results in the non-absorption of outgoing waves. Such a condition leads to inaccurate calculations or even instability. Thus, an alternative method for low-pressure discharge simulation is becoming increasingly important than ever before with the development of satellite sensors in outer space. Until now, most references have been focused on the simulation of discharge threshold [41,42,43,44,45]. To the best of our knowledge, the influence of electrical behavior in low-pressure discharge circumstances has not been investigated and developed. Such influence becomes of vital importance for satellite sensors in outer space. When such a condition happens, the performance can be recovered and adjusted by employing the outer components and circuits based on the analysis.

Here, a one-step CNDS algorithm is proposed to efficiently analyze the low-pressure discharge for satellite sensors in outer space. The ionized gas, which significantly affects the electrical behavior in the occurrence of low-pressure discharge, can be analyzed according to the Lorentz model with the PLRC method. Inside PML regions, higher order formulation is proposed based on the CNDS scheme inside Lorentz medium. Through the low-pressure discharge simulation, the proposed algorithm shows considerable accuracy and efficiency. At the boundaries of the unbounded lattice, the proposed algorithm can enhance performance by further absorbing the low-frequency waves and late-time reflections. By employing the parameters from the experiment, results indicate that the significant difference is caused by the low-pressure discharge phenomenon. Meanwhile, the experiment shows considerable agreement with the simulation, which also demonstrates the effectiveness of the proposed algorithm.

## 2. Formulation

In a complex medium, Maxwell’s equations inside the PML regions can be given in the following form as

(1a) $$j\omega D=\nabla_{s}\times H$$

(1b) $$j\omega H=-\nabla_{s}\times E$$

where D is the electric displacement of Maxwell’s equation which can be obtained by the relationship as $D\left(\omega \right)=\varepsilon_{0}\varepsilon_{r}\left(\omega \right)E\left(\omega \right)$. It has been mentioned that ionized gas caused by the low-pressure discharge is generated due to the excitation of the high power. Such condition can be expressed by the Lorentz medium, given as

(2) $$\varepsilon_{r}\left(\omega \right)=\varepsilon_{\infty }+\frac{\left(\varepsilon_{s}-\varepsilon_{\infty }\right)\omega_{0}^{2}}{\left(\omega_{0}^{2}+j2\delta \omega -\omega^{2}\right)}$$

where the parameters can be given as: $\varepsilon_{\infty }$ and $\varepsilon_{s}$ represent the relative permittivity at infinite frequency and static permittivity, $\omega_{0}$ represents the resonance radian frequency and δ represents the damping constant. The operator $\nabla_{s}$ can be obtained as

(3) $$\nabla_{s}=\hat{x}\frac{1}{S_{x}}\frac{\partial }{\partial x}+\hat{y}\frac{1}{S_{y}}\frac{\partial }{\partial y}+\hat{z}\frac{1}{S_{z}}\frac{\partial }{\partial z}$$

where $S_{\eta },\;\eta =x,y,z$ is the stretched coordinate variables. Inside the higher-order PML regions, it can be obtained by multiplying the stretched coordinate variables together into a single term, expressed as

(4) $$S_{\eta }=\left(\kappa_{\eta 1}+\frac{\sigma_{\eta 1}}{\alpha_{\eta 1}+j\omega \varepsilon_{0}}\right)\left(\kappa_{\eta 2}+\frac{\sigma_{\eta 2}}{\alpha_{\eta 2}+j\omega \varepsilon_{0}}\right)$$

where $\kappa_{\eta n},\;n=1,2$ is the positive real and $\sigma_{\eta n}$ and $\alpha_{\eta n}$ are real. According to the Z-transformation relationship, $j\omega ↔\left(1-z^{-1}\right)/\Delta t$, Maxwell’s equations can be transformed into Z-domain as

(5a) $$\frac{1-z^{-1}}{\Delta t}D_{z}=S_{x}^{-1}\left(z\right)\frac{\partial H_{y}}{\partial x}-S_{y}^{-1}\left(z\right)\frac{\partial H_{x}}{\partial y}$$

(5b) $$-\mu_{0}\frac{1-z^{-1}}{\Delta t}H_{z}=S_{x}^{-1}\left(z\right)\frac{\partial E_{y}}{\partial x}-S_{y}^{-1}\left(z\right)\frac{\partial E_{x}}{\partial y}$$

Due to the existence of massive components, components along the z-direction are chosen as examples for demonstration. The other components can be obtained according to a similar method. Inside Equation (5a,b), $S_{\eta }^{-1}\left(z\right)$ is the stretched coordinate variables in the Z-domain, given as

(6) $$S_{\eta }(z)=w_{\eta 1}w_{\eta 2}\frac{1-a_{\eta 1}z^{-1}}{1-b_{\eta 1}z^{-1}}\frac{1-a_{\eta 2}z^{-1}}{1-b_{\eta 2}z^{-1}}$$

where the coefficients can be given as

$w_{\eta n}=\frac{1}{\kappa_{\eta n}}\cdot (\frac{2}{\Delta t}+\frac{\alpha_{\eta n}}{\varepsilon_{0}})/[\frac{2}{\Delta t}+(\frac{\alpha_{\eta n}}{\varepsilon_{0}}+\frac{\sigma_{\eta n}}{\kappa_{\eta n}\varepsilon_{0}})]$, $a_{\eta n}=(\frac{2}{\Delta t}-\frac{\alpha_{\eta n}}{\varepsilon_{0}})/(\frac{2}{\Delta t}+\frac{\alpha_{\eta n}}{\varepsilon_{0}})$ and $b_{\eta n}=[\frac{2}{\Delta t}-(\frac{\alpha_{\eta n}}{\varepsilon_{0}}+\frac{\sigma_{\eta n}}{\kappa_{\eta n}\varepsilon_{0}})]/[\frac{2}{\Delta t}+(\frac{\alpha_{\eta n}}{\varepsilon_{0}}+\frac{\sigma_{\eta n}}{\kappa_{\eta n}\varepsilon_{0}})]$. By substituting Equation (6) into Equation (5a,b), one obtains

(7a) $$\frac{1-z^{-1}}{\Delta t}D_{z}=w_{x1}w_{x2}\frac{1-a_{x1}z^{-1}}{1-b_{x1}z^{-1}}\frac{1-a_{x2}z^{-1}}{1-b_{x2}z^{-1}}\frac{\partial H_{y}}{\partial x}-w_{y1}w_{y2}\frac{1-a_{y1}z^{-1}}{1-b_{y1}z^{-1}}\frac{1-a_{y2}z^{-1}}{1-b_{y2}z^{-1}}\frac{\partial H_{x}}{\partial y}$$

(7b) $$-\mu_{0}\frac{1-z^{-1}}{\Delta t}H_{z}=w_{x1}w_{x2}\frac{1-a_{x1}z^{-1}}{1-b_{x1}z^{-1}}\frac{1-a_{x2}z^{-1}}{1-b_{x2}z^{-1}}\frac{\partial E_{y}}{\partial x}-w_{y1}w_{y2}\frac{1-a_{y1}z^{-1}}{1-b_{y1}z^{-1}}\frac{1-a_{y2}z^{-1}}{1-b_{y2}z^{-1}}\frac{\partial E_{x}}{\partial y}$$

In order to update the equations inside PML regions, auxiliary variables are introduced in Equation (7a,b). According to the introduction of auxiliary variables F and G, the original equations can be given in the following form as

(8a) $$\frac{1-z^{-1}}{\Delta t}D_{z}=\left(F_{zx2}-a_{x1}z^{-1}F_{zx2}\right)-\left(F_{zy2}-a_{y1}z^{-1}F_{zy2}\right)$$

(8b) $$-\mu_{0}\frac{1-z^{-1}}{\Delta t}H_{z}=\left(G_{zx2}-a_{x1}z^{-1}G_{zx2}\right)-\left(G_{zy2}-a_{y1}z^{-1}G_{zy2}\right)$$

where the coefficients can be given as, for example,

(9a) $$F_{z\eta 1}=b_{\eta 1}z^{-1}F_{z\eta 1}+w_{\eta 1}\frac{\partial H_{\tilde{\eta }}}{\partial \eta }$$

(9b) $$F_{z\eta 2}=b_{\eta 2}z^{-1}F_{z\eta 2}+w_{\eta 2}\cdot (1-a_{\eta 2}z^{-1})F_{z\eta 1}$$

(9c) $$G_{z\eta 1}=b_{\eta 1}z^{-1}G_{z\eta 1}+w_{\eta 1}\frac{\partial E_{\tilde{\eta }}}{\partial \eta }$$

(9d) $$G_{z\eta 2}=b_{\eta 2}z^{-1}G_{z\eta 2}+w_{\eta 2}\cdot (1-a_{\eta 2}z^{-1})G_{z\eta 1}$$

where $\tilde{\eta }$ represents the rest component excepting η. For example, when calculating $E_{z}$ and $H_{z}$ components, η=x while $\tilde{\eta }=y$. By substituting the auxiliary variables into the components, results can be given as

(10a) $$\frac{1-z^{-1}}{\Delta t}D_{z}=\left[\left(b_{x1}-a_{x2}\right)F_{zx1}+\left(b_{x2}-a_{x1}\right)F_{zx2}+w_{x1}w_{x2}\frac{\partial H_{y}}{\partial x}\right]\;-\left[\left(b_{y1}-a_{y2}\right)F_{zy1}+\left(b_{y2}-a_{y1}\right)F_{zy2}+w_{y1}w_{y2}\frac{\partial H_{x}}{\partial y}\right]$$

(10b) $$\mu_{0}\frac{1-z^{-1}}{\Delta t}H_{z}=\left[\left(b_{y1}-a_{y2}\right)G_{zy1}+\left(b_{y2}-a_{y1}\right)G_{zy2}+w_{y1}w_{y2}\frac{\partial E_{x}}{\partial y}\right]\;-\left[\left(b_{x1}-a_{x2}\right)G_{zx1}+\left(b_{x2}-a_{x1}\right)G_{zx2}+w_{x1}w_{x2}\frac{\partial E_{y}}{\partial x}\right]$$

In order to update the equations including the electric displacement components, the PLRC method is introduced to analyze the relationship between the electric displacement and field components. Through the introduction of the PLRC method and CN scheme, Equation (10a,b) can be further rearranged and discretized in the FDTD domain as

(11a) $$E_{z}^{n+1}=a_{1}E_{z}^{n}+a_{2}\varphi_{z}^{n}+p_{1ex}F_{zx1}^{n}+p_{2ex}F_{zx2}^{n}+p_{3ex}\delta_{x}\left(H_{y}^{n+1}+H_{y}^{n}\right)\;-p_{1ey}F_{zy1}^{n}-p_{2ey}F_{zy2}^{n}-p_{3ey}\delta_{y}\left(H_{x}^{n+1}+H_{x}^{n}\right)$$

(11b) $$H_{z}^{n+1}=H_{z}^{n+1}+p_{1hy}G_{zy1}+p_{2hy}G_{zy2}+p_{3hy}\delta_{y}\left(E_{x}^{n+1}+E_{x}^{n}\right)\;+p_{1hx}G_{zx1}-p_{2hx}G_{zx2}-p_{3hx}\delta_{x}\left(E_{y}^{n+1}+E_{y}^{n}\right)$$

where the coefficients can be given as

$$\mathrm{PLRC}\;\mathrm{method}:a_{1}=\frac{8-2\omega_{0}^{2}\Delta t^{2}}{\left(4+4\delta \Delta t+\omega_{0}^{2}\Delta t^{2}\right)},\;a_{2}=-\frac{\left(4-4\delta \Delta t+\omega_{0}^{2}\Delta t^{2}\right)}{\left(4+4\delta \Delta t+\omega_{0}^{2}\Delta t^{2}\right)}\;$$

PML regions: $p_{1e\eta }=a_{2}\Delta t(a_{\eta 1}-b_{\eta 2})/\varepsilon_{0}$,$p_{2e\eta }=a_{2}\Delta t(a_{\eta 2}-b_{\eta 1})/\left(2\varepsilon_{0}\right)$, $p_{3e\eta }=a_{2}\Delta tw_{\eta 1}w_{\eta 2}/\varepsilon_{0}$, $p_{1h\eta }=\Delta t(a_{\eta 1}-b_{\eta 2})/\mu_{0}$, $p_{2h\eta }=\Delta t(a_{\eta 2}-b_{\eta 1})/\mu_{0}$ and $p_{3h\eta }=\Delta tw_{\eta 1}w_{\eta 2}/\left(2\mu_{0}\right)$. It can be observed from Equation (11a,b) that components at the time step of n + 1 and n exist at both sides of the equations resulting in the formation of coupled equations. Although it can be solved according to the original CN scheme, large sparse matrices must be solved at each time step resulting in much more expensive computation. In order to alleviate such conditions, approximate CN algorithms are proposed to avoid the calculation of sparse matrices. Among approximate CN algorithms in three dimensions, CNDS algorithm shows considerable accuracy and efficiency. According to the CNDS algorithm, the entire updated equations can be given in the matrix form as

(12) $$\left(I-D_{1}-D_{2}\right)\Phi^{n+1}=\left(I_{1}+D_{1}+D_{2}\right)\Phi^{n}+A^{n}$$

where I is the identity matrix with the dimensions of 6×6, $\Phi ={\left[E_{x},E_{y},E_{z},H_{x},H_{y},H_{z}\right]}^{T}$ and A is the other components at the right side of Equation (11a,b); matrices of $D_{1}$ and $D_{2}$ can be obtained as

$$D_{1}=\left[\begin{array}{cccccc} 0 & 0 & 0 & 0 & -p_{3ez}\delta_{z} & 0\\ 0 & 0 & 0 & 0 & 0 & -p_{3ex}\delta_{x}\\ 0 & 0 & 0 & -p_{3ey}\delta_{y} & 0 & 0\\ 0 & 0 & -p_{3hy}\delta_{y} & 0 & 0 & 0\\ -p_{3hz}\delta_{z} & 0 & 0 & 0 & 0 & 0\\ 0 & -p_{3hx}\delta_{x} & 0 & 0 & 0 & 0 \end{array}\right]$$

$$D_{2}=\left[\begin{array}{cccccc} 0 & 0 & 0 & 0 & 0 & p_{3ey}\delta_{y}\\ 0 & 0 & 0 & p_{3ez}\delta_{z} & 0 & 0\\ 0 & 0 & 0 & 0 & p_{3ex}\delta_{x} & 0\\ 0 & p_{3hz}\delta_{z} & 0 & 0 & 0 & 0\\ 0 & 0 & p_{3hx}\delta_{x} & 0 & 0 & 0\\ p_{3hy}\delta_{y} & 0 & 0 & 0 & 0 & 0 \end{array}\right]$$

In order to decouple the coupled equations, $D_{1}D_{2}\Phi^{n+1}$ and $D_{1}D_{2}\Phi^{n}$ are added at both sides of the equations. According to the factoring factorization method, Equation (12) can be given as

(13a) $$\left(I-D_{1}\right)\Phi^{*}=\left(I_{1}+D_{1}+2D_{2}\right)\Phi^{n}+A^{n}$$

(13b) $$\left(I-D_{2}\right)\Phi^{n+1}=\Phi^{*}-D_{2}\Phi^{n}$$

According to the CNDS algorithm, Maxwell’s equations can be updated by referring to Equation (13a,b). It can be given in the following forms, where one obtains

(14a) $$E_{z}^{*}=a_{1}E_{z}^{n}+a_{2}\varphi_{z}^{n}+p_{1ex}F_{zx2}^{n}+p_{2ex}F_{zx1}^{n}-p_{1ey}F_{zy2}^{n}-p_{2ey}F_{zy1}^{n}-p_{3ey}\delta_{y}\left(H_{x}^{*}+H_{x}^{n}\right)+2p_{3ex}\delta_{x}H_{y}^{n}$$

(14b) $$H_{z}^{*}=H_{z}^{n}+p_{1hy}G_{zy1}^{n}+p_{2hy}G_{zy2}^{n}-p_{1hx}G_{zx1}^{n}-p_{2hx}G_{zx2}^{n}-p_{3hx}\partial_{x}\left(E_{y}^{*}+E_{y}^{n}\right)+2p_{3hy}\partial_{y}E_{x}^{n}$$

(14c) $$E_{z}^{n+1}=E_{z}^{*}+p_{3ex}\partial_{x}\left(H_{y}^{n+1}+H_{y}^{n}\right)$$

(14d) $$H_{z}^{n+1}=H_{z}^{*}+p_{3hy}\partial_{y}\left(E_{x}^{n+1}+E_{x}^{n}\right)$$

In order to eliminate the mid-terms, Equation (14d) is substituted into Equation (14a) as

$$\begin{array}{l} \left(1-p_{3ey}p_{3hy}\delta_{2y}\right)E_{z}^{*}=\left(a_{1}+p_{3ey}p_{3hy}\delta_{2y}\right)E_{z}^{n}+a_{2}\varphi_{z}^{n}\\ +p_{1ex}F_{zx2}^{n}+p_{2ex}F_{zx1}^{n}-p_{1ey}F_{zy2}^{n}-p_{2ey}F_{zy1}^{n} \end{array}$$

$$-p_{1hz}p_{3ey}\delta_{y}G_{xz2}^{n}-p_{2hz}p_{3ey}\delta_{y}G_{xz1}^{n}+p_{1hy}p_{3ey}\delta_{y}G_{xy2}^{n}+p_{2hy}p_{3ey}\delta_{y}G_{xy1}^{n}$$

(15) $$-2p_{3ey}\delta_{y}H_{x}^{n}+2p_{3ex}\delta_{x}H_{y}^{n}-2p_{3hz}p_{3ey}\delta_{yz}E_{y}^{n}$$

To eliminate components at the time step of n+1, Equation (14d) is substituted into Equation (14a) as

$$\begin{array}{l} \left(1-p_{3ex}p_{3hx}\delta_{2x}\right)E_{z}^{n+1}=E_{z}^{*}-p_{3ex}p_{3hx}\delta_{2x}\left(E_{x}^{*}+E_{x}^{n}\right)\\ +2p_{3ex}\delta_{x}H_{y}^{n}+p_{3ex}p_{3hx}\delta_{2x}E_{z}^{n}+p_{1hx}p_{3ex}\delta_{x}G_{yx2}^{n} \end{array}$$

(16) $$+p_{2hx}p_{3ex}\delta_{x}G_{yx1}^{n}-p_{1hz}p_{3ex}\delta_{x}G_{yz2}^{n}-p_{2hz}p_{3ex}\delta_{x}G_{yz1}^{n}$$

According to the PLRC method, one obtains

(17) $$\varphi_{z}^{n+1}=a_{1}\varphi_{z}^{n}+a_{2}\varphi_{z}^{n-1}+a_{3}E_{z}^{n+1}+2a_{3}E_{z}^{n}+a_{3}E_{z}^{n-1}$$

where $a_{3}=\frac{\Delta \varepsilon \omega_{0}^{2}\Delta t^{2}}{\left(4+4\delta \Delta t+\omega_{0}^{2}\Delta t^{2}\right)}$

It can be observed that tri-diagonal matrices are formed at the left sides of Equations (15) and (16) which can be directly solved according to the Thomas algorithm. Each component needs to solve two matrices during the update iteration. Thus, the CNDS algorithm solves six matrices during a single iteration which results in the improvement of efficiency compared with the CNAFS algorithm.

## 3. Numerical Results and Experiments

**Figure 1:**
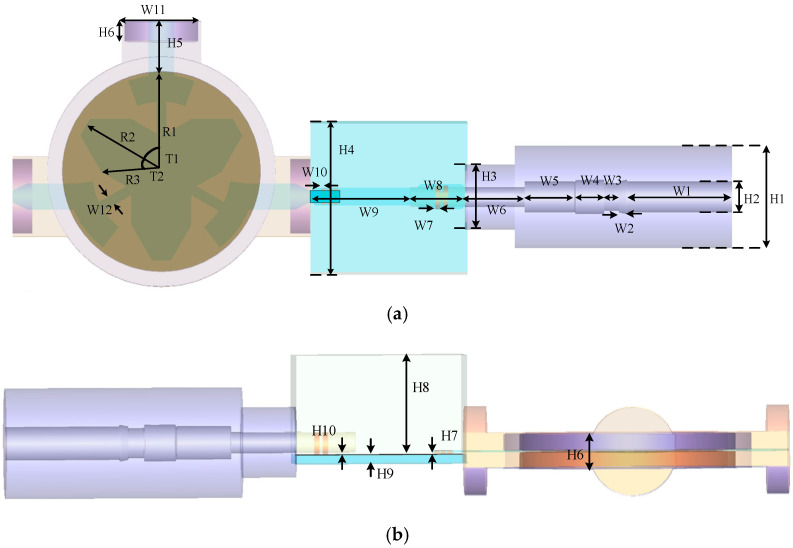
The sketch picture of the satellite sensors (**a**) top view (**b**) front view.

In order to demonstrate the effectiveness of the proposed algorithm and analyze the low-pressure discharge phenomenon for the satellite sensors in outer space, a satellite sensors system which is composed of a waveguide, transmission line, connector and circulator is employed as an example for demonstration. So far, several techniques are developed based on the Lorentz model which can be extensively employed in the analysis of low-pressure discharge phenomenon including the conventional explicit FDTD algorithm-based CFS-PML (FDTD-PML) in [46] and ADI algorithm-based CFS-PML (ADI-PML) in [47]. Due to the limitation of medium-dependent characteristics of the FDTD algorithm, massive existing algorithms cannot be directly extended into the simulation of the Lorentz model. Thus, these algorithms cannot be employed in the low-pressure discharge phenomenon. Hence, FDTD-PML and ADI-PML algorithms are employed in the comparison and demonstration. The proposed scheme is denoted as CNDS-PML to simplify the demonstration. Figure 1 shows the sketch picture of the satellite sensors system from the top view and front view.

From Figure 1a, it can be observed that the satellite sensors system is composed of a circulator, transmission line, waveguide and connector from the left to the right. The circulator can be regarded as the patched component and is composed of the vertical magnetized ferrite material with the parameter of $\varepsilon_{r}=14.9$. The substrate of the ferrite is composed of Teflon dielectric plate with a parameter of $\varepsilon_{r}=2.02$. The patch on the top surface of the ferrite material is made up of the metal gold. Three ports are included in the circulator model which can be regarded as a combination of a circle with a radius of 4 mm and a rectangle of 8×3 mm. The middle of the structure is the transmission line component with a metal connector. Inside the transmission line model, Rogers RO4003 with the parameter of 3.55 is employed as the substrate material. The bands which are located just above the substrate are also made up of metallic gold. The right side of the structure is the coaxial waveguide structure. Inside the waveguide model, Teflon dielectric bulk and metal center which can be expressed by the perfectly electronic conductor (PEC) are employed. The detailed parameters of the entire model are listed in Table 1 with the unit of a millimeter (mm).

**Figure 2:**
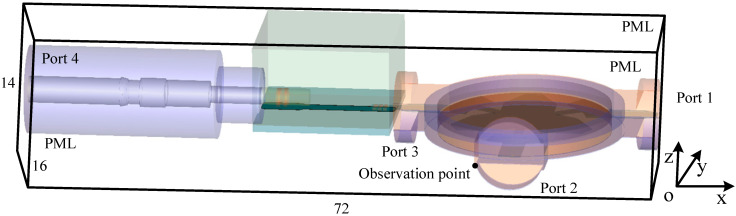
The sketch picture of the satellite sensors inside the FDTD computational domain.

Figure 2 shows the sketch picture of the computational domain in the FDTD simulation. The entire rectangle domain has the dimensions of 72×16×14 mm in each direction. The sensor is located in the middle of the domain. Four ports are employed at the positions of the left, middle, front and right of the domain to evaluate the electrical behavior. Port 1, port 2 and port 3 hold the same dimensions and shapes which can be regarded as a combination of circle and rectangle models. Port 4 holds the circle model with a radius of 4.25 mm. The excitation source is the plane source that holds the dimensions of the port. Here, the source is excited in port 1 which can be regarded as the combination of a circle with a radius of 4 mm and a rectangle of 8×3 mm of the yOz plane is employed as an example for demonstration. The Gaussian pulse source with the maximum frequency of 2 GHz is employed to excite the model. The observation point is located at the corner of port 2 with a distance of 1 cell beside the PML regions. At the boundaries of the domain, 10-cell-PML regions are employed to terminate unbounded lattices and reflect outgoing waves. The parameters inside PML regions are selected for the best absorbing performance both in the time domain and frequency domain. The parameters inside the proposed PML regions are $\kappa_{\eta 1}=11$, $\alpha_{\eta 1}=1.4$, $m_{\eta 1}=4$, $\sigma_{\eta 1\_\max}=0.01\sigma_{\eta 1\_opt}$, $\kappa_{\eta 2}=12$, $\alpha_{\eta 2}=3.1$, $m_{\eta 2}=1$ and $\sigma_{\eta 2\_\max}=0.01\sigma_{\eta 2\_opt}$, where $\sigma_{\eta n\_opt}=(m_{\eta n}+1)/(150\pi \Delta \eta )$. The parameters of the other schemes are chosen as $\kappa_{\eta }=36$, $\alpha_{\eta 1}=0.68$, $m_{\eta }=2$ and $\sigma_{\eta \_\max}=0.9\sigma_{\eta \_opt}$.

**Figure 3:**
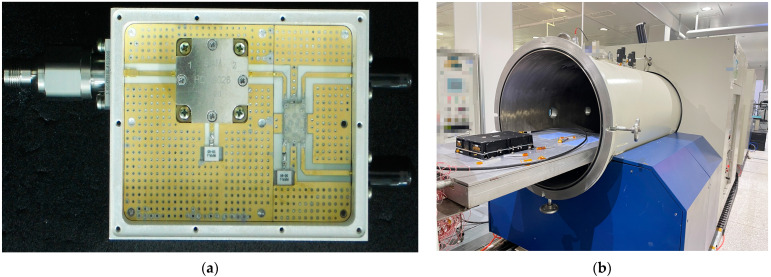
(**a**) The manufactured filter sensors for satellite (**b**) low-pressure discharge measurement system.

Figure 3 shows the picture of the manufactured satellite sensors system inside the metal cavity and the experiment environment. Figure 3a represents the manufactured satellite sensors system with three outer ports, which correspond to port 1, port 3 and port 4, as shown in Figure 2. SMA connector is employed at port 1 to excite the entire component. In the middle of the component, the patched circulator is located under the extremely thin metal surface. Port 2 is located at the bottom of the circulator. On the right side of the circulator, the transmission line model is located under the metal surface. The upper port corresponds to port 3. The coaxial waveguide model is located at the right side of the transmission line, whose signal can be measured from the right bottom port, which corresponds to port 4. Figure 3b shows the experiment which can evaluate the low-pressure discharge phenomenon and analyze the environment in outer space. Components are located inside the metal cavity. Low-pressure, large-range-variation temperatures can occur inside the cavity, which can accurately simulate the environment in outer space. Meanwhile, through the employment of the system, parameters of the low-pressure discharge can be measured.

With the occurrence of a low-pressure discharge phenomenon, the gas inside the sensors devices is ionized by the high power resulting in the formation of plasma. Through the experiment, plasma can be expressed by the Lorentz media with the parameters of $\varepsilon_{\infty }=1$, $\varepsilon_{s}=2.25$, σ=0 S/m, $\omega_{0}=4\times 10^{16}\;\mathrm{rad}/s$ and $\delta =0.28\times 10^{16}{\;s}^{-1}$. Furthermore, when the sensors system works with non-dischargement circumstances, the computational domain can be regarded as filling with air.

**Figure 4:**
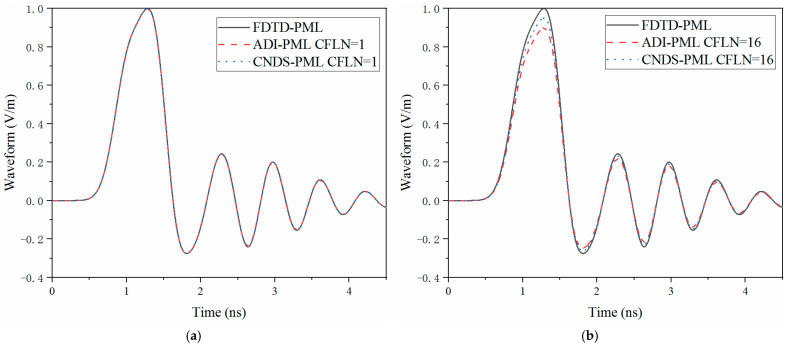
The waveform obtained by different PML algorithms and CFLNs at the observation point in the time domain (**a**) FDTD-PML, ADI-PML and CNDS-PML with CFLN = 1. (**b**) FDTD-PML, ADI-PML and CNDS-PML with CFLN = 16.

In the unconditionally stable algorithms, the mesh size of the calculation can be chosen according to the accuracy rather than the CFL condition. Here, mesh size can be chosen as Δx=Δy=Δz=Δ=0.1 mm. Thus, the entire computational domain can be discretized as 720Δx×160Δy×140Δz according to Yee’s grid. The maximum time step of the conventional FDTD algorithm $\Delta t_{\max}^{FDTD}$ according to the CFL condition can be obtained as 0.29 ps. The CFL number (CFLN) is defined as $CFLN=\Delta t/\Delta t_{\max}^{FDTD}$, where Δt is the time step in the unconditionally stable algorithm. It has been testified that CFLN = 16 holds the best compromise between accuracy and efficiency [48]. Thus, such a circumstance is employed as an example for demonstration. The calculation accuracy in the time domain can be demonstrated by the time domain waveform. Figure 4 shows the waveform at the observation point obtained by different algorithms and CFLNs in the time domain.

Through Figure 4a, it can be observed that these curves are almost overlapped. Such a condition indicates that these algorithms hold the same calculation accuracy with CFLN = 1. As shown in Figure 4b, curves show shifting compared with those obtained by CFLN = 1. The condition indicates the calculation accuracy degenerates with the enlargement of CFLNs. The reason is that the numerical dispersion increases with the enlargement of the time step, which results in the degeneration of calculation accuracy. Among unconditionally stable algorithms, it can be observed that the proposed CNDS-PML algorithm shows less shifting compared with the implicit ADI-PML scheme. Such a condition indicates the proposed algorithm behaviors less numerical dispersion and better accuracy compared with the existing implicit scheme.

**Figure 5:**
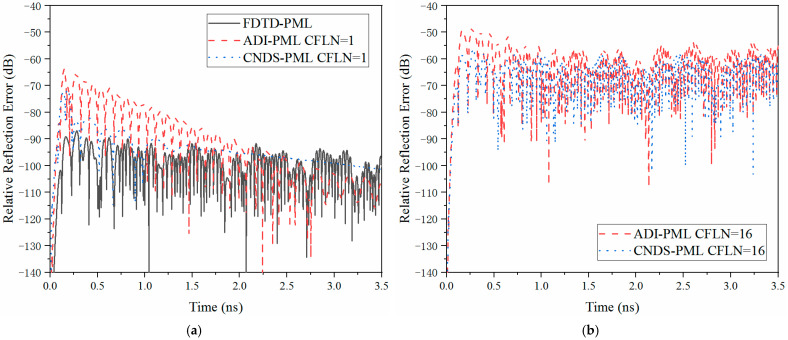
The relative reflection error obtained by different PML algorithms and CFLNs at the observation point in the time domain (**a**) FDTD-PML, ADI-PML and CNDS-PML with CFLN = 1. (**b**) FDTD-PML, ADI-PML and CNDS-PML with CFLN = 16.

In order to further evaluate the absorbing performance inside the PML regions, relative reflection error in the time domain is employed, which can be defined as

(18) $$R_{dB}\left(t\right)=20\log_{10}\left[\left|E_{z}^{t}\left(t\right)-E_{z}^{r}\left(t\right)\right|/\left|\max\{E_{z}^{r}\left(t\right)\}\right|\right]$$

where $E_{z}^{t}\left(t\right)$ is the test solution which can be obtained directly from the observation point, $E_{z}^{r}\left(t\right)$ is the reference solution which can be obtained with enlarged computational domain and thicker PML regions. Due to the employment of thicker PML regions with 128 cells and an enlarged domain with 20 times, the reflection wave can be ignored at the observation point without changing the relative position between the source and the observation point. Figure 5 demonstrates the relative reflection error obtained by different PML algorithms and CFLNs in the time domain.

As can be observed from Figure 5a that the time-explicit conventional FDTD-PML holds the best absorbing performance due to the non-calculation of matrices. Such condition results in the best calculation accuracy among these introduced algorithms. The performance of the implicit algorithms decreases due to the calculation of matrices at each time step resulting in the degeneration of accuracy and efficiency with smaller time steps. Compared with the existing implicit ADI-PML algorithm, the proposed algorithm can receive better absorption due to the improvement of the calculation accuracy. From Figure 5b, it can be observed that the absorption decreases significantly with the enlargement of CFLNs due to the enlargement of numerical dispersion. It can still conclude that the proposed CNDS*PML scheme receives better performance and absorption compared with the existing implicit ADI-PML scheme. Although the absorption decreases with CFLN = 16, it still maintains a considerable level, which can be employed in practical engineering (usually regarded as −40 dB as a standard) [48]. The effectiveness of the calculation can also be reflected by the memory consumption and simulation duration occupied by different algorithms and CFLNs, as shown in Table 2.

As can be observed from Table 2, the simulation duration and memory consumption of the implicit algorithm becomes larger compared with the conventional explicit scheme. The reason is that the implicit algorithms solve six tridiagonal matrices and more coefficients at each time step. The calculation of matrices consumes much more resources on the simulation duration. The increment of coefficients also results in the degeneration of efficiency and enlargement of memory consumption. Compared with memory consumption, the development of computational electromagnetics mainly focuses on efficiency.

As can be observed, the efficiency can be enhanced by employing larger CFLNs, shown in the last two columns of Table 2. The enlargement of CFLN results in the increment of a simulation time step. In such circumstances, the simulation duration can be shortened according to the decrement of the total simulation iteration step. Thus, in an unconditionally stable algorithm, a large CFLN which leads to an enlarged time step can receive better efficiency compared with the smaller one. Compared with the existing ADI-PML scheme, the proposed CNDS-PML shows considerable efficiency and memory consumption. With CFLN = 16, the proposed implicit algorithm shows significant improvement in simulation duration compared with the other algorithms. Most importantly, it can obtain better memory consumption and efficiency compared with the existing ADI-PML algorithm. Such a condition indicates the improvement of effectiveness from the aspect of simulation duration and memory consumption.

**Figure 6:**
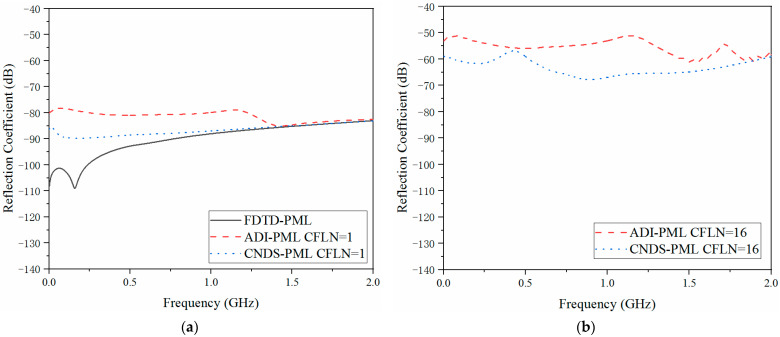
The reflection coefficient obtained by different PML algorithms and CFLNs at the observation point in the frequency domain (**a**) FDTD-PML, ADI-PML and CNDS-PML with CFLN = 1. (**b**) FDTD-PML, ADI-PML and CNDS-PML with CFLN = 16.

The absorption inside the PML regions cannot only be reflected by the relative reflection error in the time domain but also be evaluated by the reflection coefficient in the frequency domain, which can be defined as

(19) $$R_{dB}\left(f\right)=20\log_{10}\left|FFT\left\{E_{z}^{t}\left(t\right)-E_{z}^{r}\left(t\right)\right\}/FFT\left\{E_{z}^{t}\left(t\right)\right\}\right|$$

where the manipulation operator FFT denotes the Fourier transformation. Figure 6 shows the reflection coefficient obtained by different PML algorithms and CFLNs in the frequency domain.

Through Figure 6a, we can draw the same conclusion that the conventional explicit FDTD-PML algorithm still holds the best absorption in the entire frequency domain simulation due to the non-matrices calculation. Due to the solution of matrices at each time step, absorption degenerates in the implicit algorithms. Among the implicit schemes, the proposed CNDS-PML algorithm receives a better reflection coefficient compared with the existing implicit ADI-PML algorithm. From Figure 6b, the absorption decreases with the enlargement of the time step due to the enlargement of numerical dispersion, which corresponds to the improvement of the reflection coefficient in the frequency domain. Compared with the existing implicit ADI-PML algorithm, the proposed scheme still holds better absorption in the entire frequency band. Meanwhile, the wave reflection at low frequency can be decreased by employing the higher-order formulation. Such a condition proves that the low-frequency propagation waves can be further absorbed. In summary, the employment of the higher-order formulation enhances the absorption both in the time domain and frequency domain. However, such a condition increases the simulation duration and memory consumption during the whole simulation. Such a condition demonstrates that the higher-order formulation can be regarded as a compromise between efficiency and absorption.

**Figure 7:**
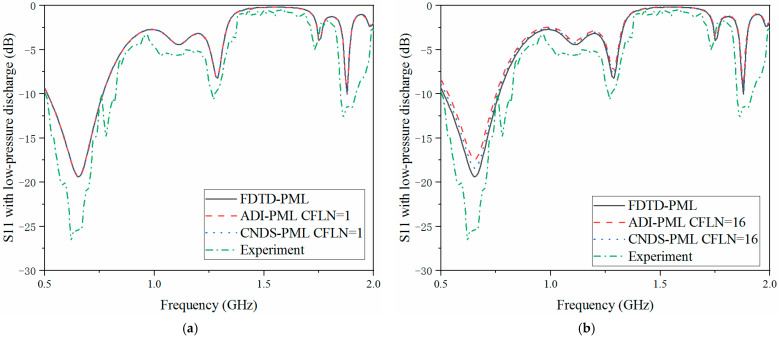
S11 parameter with low-pressure discharge obtained by different PML algorithms and CFLNs in the frequency domain (**a**) FDTD-PML, ADI-PML and CNDS-PML with CFLN = 1. (**b**) FDTD-PML, ADI-PML and CNDS-PML with CFLN = 16.

**Figure 8:**
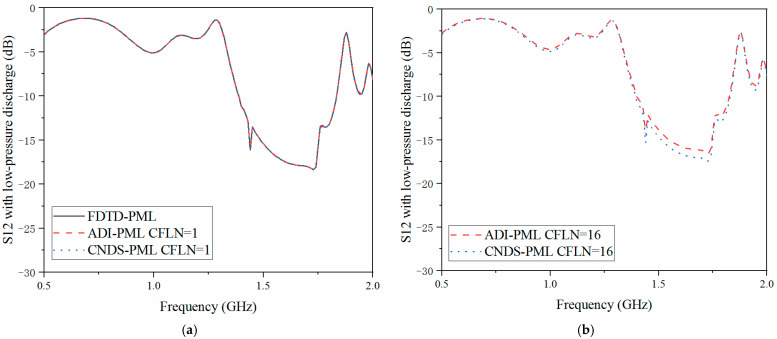
S12 parameter with low-pressure discharge obtained by different PML algorithms and CFLNs in the frequency domain (**a**) FDTD-PML, ADI-PML and CNDS-PML with CFLN = 1. (**b**) FDTD-PML, ADI-PML and CNDS-PML with CFLN = 16.

**Figure 9:**
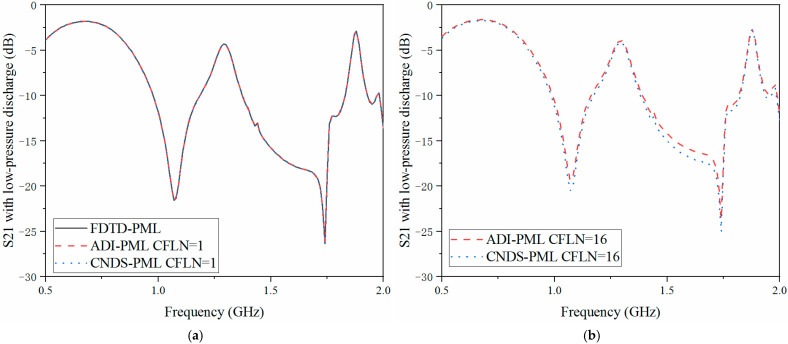
S21 parameter with low-pressure discharge obtained by different PML algorithms and CFLNs in the frequency domain (**a**) FDTD-PML, ADI-PML and CNDS-PML with CFLN = 1. (**b**) FDTD-PML, ADI-PML and CNDS-PML with CFLN = 16.

The scattering parameters can be regarded as the important parameters during the simulation and sensors system. The return loss (S11), transmission coefficient (S21) and isolation (S12) are employed for demonstration during the simulation. Here, calculation accuracy and absorption can also be reflected by the scattering parameters in the frequency, as shown in Figure 7, Figure 8 and Figure 9. Meanwhile, in order to demonstrate the effectiveness of the proposed algorithm, experiment results are included. Inside the cavity, the measurement of the excitation signal and echo wave signal depends on the same probe. Thus, S11 can be obtained from the experiment, as shown in Figure 7. 

As shown in Figure 7a, the curves are almost overlapped with CFLN = 1. This condition indicates these algorithms hold the same calculation accuracy during the entire frequency simulation. With the increment of CFLN and time step, curves obtained by implicit algorithms show shifting compared with these algorithms with CFLN = 1. This condition indicates a decrement in calculation accuracy due to the increment of numerical dispersion. Among implicit algorithms, the proposed CNDS-PML algorithm shows better performance compared with the implicit ADI-PML algorithm, shown in Figure 7b. As can be demonstrated from the experiment results, it shows considerable agreement with the simulation method. The condition shows the effectiveness of the proposed algorithm in the simulation of the low-pressure discharge phenomenon. Figure 8 and Figure 9 show the S12 and S21 parameters obtained by different PML algorithms and CFLNs in the frequency domain with low-pressure discharge phenomenon, respectively.

**Figure 10:**
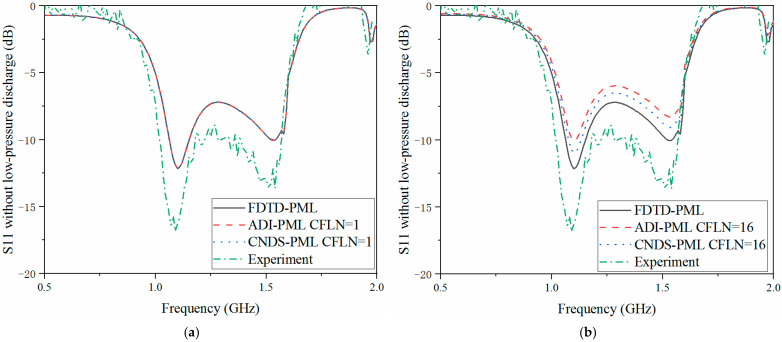
S11 parameter without low-pressure discharge obtained by different PML algorithms and CFLNs in the frequency domain (**a**) FDTD-PML, ADI-PML and CNDS-PML with CFLN = 1. (**b**) FDTD-PML, ADI-PML and CNDS-PML with CFLN = 16.

**Figure 11:**
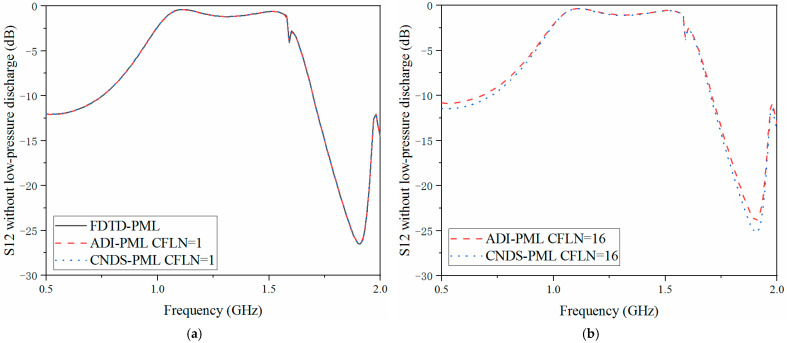
S12 parameter without low-pressure discharge obtained by different PML algorithms and CFLNs in the frequency domain (**a**) FDTD-PML, ADI-PML and CNDS-PML with CFLN = 1. (**b**) FDTD-PML, ADI-PML and CNDS-PML with CFLN = 16.

**Figure 12:**
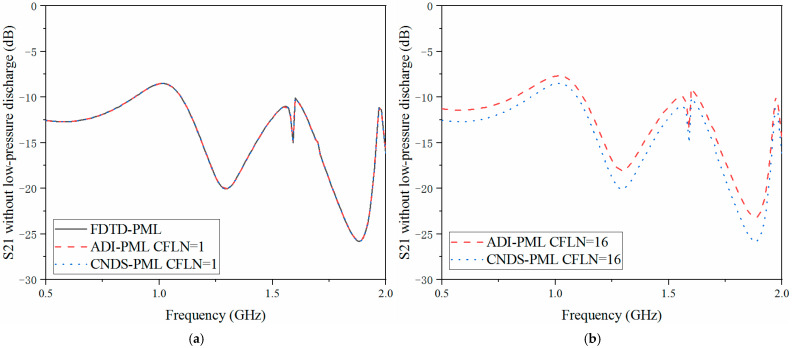
S21 parameter without low-pressure discharge obtained by different PML algorithms and CFLNs in the frequency domain (**a**) FDTD-PML, ADI-PML and CNDS-PML with CFLN = 1. (**b**) FDTD-PML, ADI-PML and CNDS-PML with CFLN = 16.

As shown in Figure 8a and Figure 9a that these curves are almost overlapped with CFLN = 1. This condition indicates they hold a similar accuracy with lower CFLNs. Through Figure 8b and Figure 9b, it can be observed that the curves show shifting compared with those with CFLN = 1 due to the enlargement of numerical dispersion and decrement of numerical accuracy with larger CFLNs. However, the proposed algorithm can still receive considerable performance with larger CFLNs. Meanwhile, it still shows considerable performance compared with existed implicit ADI-PML algorithm. Figure 10, Figure 11 and Figure 12 demonstrate the electrical behavior without low-pressure discharge, which can be regarded as filled with a vacuum at the rest of the computational domain. It can be observed that the scattering parameters show significant variation with the occurrence of the low-pressure discharge phenomenon. The occurrence of low-pressure discharge significantly affects the entire electrical behavior in the entire frequency band. Such a condition results in the failure of the satellite sensors system in outer space. Meanwhile, experiment results of the S11 parameter without the low-pressure discharge phenomenon are also considered. It can be observed that the simulation and experiment results show considerable agreement. The condition demonstrates that the proposed algorithm is efficient in practical engineering.

## 4. Conclusions

Here, an alternative algorithm is proposed to evaluate the low-pressure discharge phenomenon for the satellite sensors in outer space. For the simulation of the low-pressure discharge phenomenon, Lorentz medium is employed to accurately evaluate the electrical behavior. To be more specific, the CNDS algorithm is modified based on the higher-order formulation in the PML regions and the PLRC method in the Lorentz medium. Parameters of the Lorentz model with the occurrence of the low-pressure discharge phenomenon can be measured through the experiment. Through these parameters, electrical behavior can be evaluated by employing the proposed algorithm. It can be observed from the results that the proposed algorithm shows considerable accuracy and efficiency in the low-pressure discharge evaluation compared with the other algorithms. The higher-order formulation shows enhanced absorption at the boundaries of the domains. As can be compared between the simulation and experiment, the occurrence of low-pressure discharge phenomenon significantly affects the behavior of the sensors system. In future work, several aspects can be further investigated: (1) the proposed algorithm can be developed into the simulation of the other phenomenon, including the multipactor and so on. (2) The conformal technique can be developed according to the unconditionally stable algorithm to efficiently solve the curve structures. (3) Multi-physics problems can be considered to analyze the multi-field coupled circumstances.

## Figures and Tables

**Table 1:** The detailed parameters of the satellite sensors model (Unit: mm).

Parameter	Value	Parameter	Value
W1	10	W2	1
W3	2	W4	3
W5	5	W6	6
W7	0.6	W8	6
W9	10	W10	0.5
W11	8	W12	1
H1	9.5	H2	3
H3	6.5	H4	15
H5	5	H6	3
H7	0.01	H8	9
H9	0.8	H10	0.01
T1	45°	T2	30°
R1	10	R2	8
R3	6.5	Unit: mm	

**Table 2:** The computational duration, consumption memory, iteration step, memory increment and time reduction obtained by different PML algorithms and CFLNs.

PML Algorithm	CFLN	Steps	Memory (G)	Memory Increment (%)	Time (m)	Time Reduction (%)
FDTD-PML	1	65,536	0.7	-	22.1	-
ADI-PML	1	65,536	2.0	−185.7	107.4	−384.2
CNDS-PML	1	65,536	1.6	−157.1	90.6	−310.0
ADI-PML	16	4096	2.0	−185.7	10.3	53.4
CNDS-PML	16	4096	1.6	−157.1	6.9	68.3

## Data Availability

The data presented in this study are available on request from the corresponding author. The data are not publicly available due to the privacy of research participants.
